# The link between periodontitis and atherosclerotic cardiovascular disease in non-Hispanic White adults: NHANES 1999 to 2014

**DOI:** 10.1371/journal.pone.0321220

**Published:** 2025-04-29

**Authors:** Qingna Song, Hongyan Zhang, Yuan Su, Jukun Song

**Affiliations:** 1 Department of Intensive Care Unit, The Affilated Hospital of Qingdao University, Qingdao, China; 2 Department of Nursing Department, The Affilated Hospital of QingDao University, Qingdao, China; 3 Department of Oral and MaxillofIcial Surgery, The Affiliated Stomatological Hospital of Guizhou Medical University, Guiyang, China; Federal University of Minas Gerais: Universidade Federal de Minas Gerais, BRAZIL

## Abstract

**Background:**

Limited research exists on the association between periodontitis and Atherosclerotic Cardiovascular Disease (ASCVD) in American adults. Chronic inflammation from periodontitis may elevate ASCVD risk by promoting systemic inflammation and endothelial dysfunction, supporting the plausibility of this relationship. Therefore, we utilized data from the National Health and Nutrition Examination Survey (NHANES) database to investigate this association.

**Methods:**

We conducted a cross-sectional study using NHANES data from 1999–2004 and 2009–2014, including 5,380 individuals from the United States. Tooth loss and periodontitis were assessed through full-mouth periodontal examinations. The 10-year ASCVD risk was calculated based on the 2013 American College of Cardiology (ACC) and American Heart Association (AHA) recommendations. We employed univariate and multivariable logistic regression models, as well as subgroup and interaction analyses.

**Results:**

Among participants, 74.96% experienced tooth loss, 15.99% had moderate/severe periodontitis, and the average age was 59.64 years. ASCVD was found to be associated with moderate/severe periodontitis (OR 1.24, 95% CI 1.01–1.52, P= 0.0411) and tooth loss (OR 1.16, 95% CI 1.09–1.39, P= 0.0248) in logistic regression models after full adjustment.

**Conclusion:**

Our study establishes a significant link between moderate/severe periodontitis and the increased risk of ASCVD over a decade in American adults aged 40–79 years.

## Introduction

Periodontitis is a prevalent inflammatory condition that primarily affects the supporting structures of the teeth, including periodontal ligaments and alveolar bones [1]. This condition represents a significant public health burden, with 42% of adults affected in its moderate form and 7.8% in its severe form [2]. Importantly, periodontitis is associated with a range of systemic health issues, particularly cardiovascular disease (CVD) [3,4].

Recent epidemiological studies have explored the relationship between periodontitis and atherosclerotic cardiovascular disease (ASCVD), suggesting that individuals with severe periodontitis are at an increased risk of developing ASCVD [5–7]. ASCVD is a complex disease characterized by the accumulation of fibrofatty plaques in the arterial walls, which can lead to the majority of heart attacks and strokes [8]. According to the American Heart Association (AHA), the prevalence of CVD has risen significantly in the United States over recent decades, affecting 9.3% of adults by 2019 [9]. Despite the complexities of its pathogenesis, inflammation is a key factor at every stage of ASCVD development.

Given the observed association between periodontitis and atherosclerosis, we hypothesize that periodontitis may contribute to an elevated risk of cardiovascular events, as reflected in the ASCVD risk score, particularly among non-Hispanic white adults. The aim of this study is to investigate this potential correlation and assess its implications for clinical practice.

## Materials and methods

### Study participants

The National Health and Nutrition Examination Survey (NHANES) is a cross-sectional survey that assesses the health and nutritional status of the U.S. population. The study includes individuals of all age groups across the United States, aiming to identify the prevalence and potential causes of major illnesses. To ensure a representative sample, participants are carefully selected to reflect the demographic distribution of the U.S. population. The findings from NHANES are used to analyze the prevalence and potential causes of significant health conditions. The Institutional Review Board of the National Center for Health Statistics has approved all cycles of the NHANES protocol [10]. The website of the Centers for Disease Control and Prevention (CDC) (https://wwwn.cdc.gov/nchs/nhanes/Default.aspx) offers unrestricted data access.

### Ethics approval and consent to participate

NHANES protocol approved by NCHS Research Ethics Review Board, and obtained informed consent from all participants (https://www.cdc.gov/nchs/nhanes/irba98.htm). And all methods were performed in accordance with the Declaration of Helsinki.

### Study variables

#### Assessment of periodontitis.

This study aims to evaluate periodontitis using NHANES data collected between 2009 and 2014. Participants eligible for periodontal examination were adults aged 30–90 years who attended the Mobile Examination Center and had at least six teeth present. Additionally, participants with incomplete dental records or those who did not consent to the examination were excluded. The dental examiner conducted a thorough examination of the entire mouth at the Mobile Examination Center, which involved assessing clinical attachment loss (CAL) and probing depth (PD). The Centers for Disease Control and Prevention (CDC) and the American Periodontal Association (AAP) use the PD and CAL results to categorize the severity of periodontitis into four classifications: none, mild, moderate, and severe [11]. Specifically, severe periodontitis was characterized by a PD of 5 mm or more in at least one interproximal location and a CAL of 6 mm or more in two or more interproximal locations, excluding the same tooth. On the other hand, moderate periodontitis was identified as a PD of 5 mm or more in two or more interproximal locations with a CAL ranging from 4–6 mm in two or more interproximal locations, excluding the same tooth. Mild periodontitis is identified by a minimum PD of 4 mm in two or more interproximal regions (or a minimum PD of 5 mm in one region) and CAL measuring between 3 mm and less than 4 mm in two or more interproximal regions, excluding the identical tooth. Participants were categorized into two groups based on their periodontal condition: those without/mild periodontitis and those with moderate/severe periodontitis [12,13].

#### Assessment of tooth loss.

This study utilized NHANES data from 1999 to 2014, with the number of lost teeth serving as a secondary measure. Third molars were excluded from the analysis. The count included dental implant replacements and edentulous sites (sites without teeth), ranging from zero (for edentulous individuals) to 28 teeth. Missing teeth were recorded both categorically—0–4 teeth lost, 5–8 teeth lost, 9–27 teeth lost, and 28 teeth lost (edentulous)—and as a continuous variable.

#### Assessment of 10-year ASCVD risk.

The 2013 ACC/AHA guidelines evaluated the likelihood of experiencing a major ASCVD event within a decade for individuals between the ages of 40 and 79 who are non-Hispanic whites. This assessment utilized the 2013 Pooled Cohort Equations (PCE) [14]. The equation takes into account factors including race, gender, age, untreated or treated systolic blood pressure, overall cholesterol levels, levels of high-density lipoprotein cholesterol, the existence of diabetes, and smoking patterns. To calculate the 10-year probability of ASCVD, utilize the subsequent equation: the 10-year probability of 10-year risk of ASCVD = 1− S_10_×e^(IndX’B−MeanX’B).^ Here, S10 represents the survival rate after 10 years, IndX’B denotes the individual sum, and Mean X’B refers to the overall mean sum specific to race and sex. The risk of ASCVD over 10 years can be classified as low risk (risk <7.5%) or high risk (risk ≥7.5%), and it is a variable that varies continuously [14].

### Other covariates

As part of the initial screening, participants provided self-reported demographic details such as their age, gender, marital status, and educational attainment. The household’s income was calculated according to their Poverty Income Ratio (PIR) values [15]. The education levels were categorized as less than ninth grade, ninth through eleventh grade, high school graduation, partial college or associate (AA) degree, and completion of college or beyond. To calculate the body mass index (BMI), the body mass (measured in kilograms) is divided by the height (measured in meters) squared [16]. Participants’ BMI was categorized into two groups based on established criteria: Normal weight: BMI between 18.5 and 24.9 (18.5 ≤ BMI < 24.9); Overweight/Obese: BMI of 25 or higher (BMI ≥ 25). To classify smoking status, individuals were categorized into three groups: never-smokers, current smokers, and former smokers. Never smokers were defined as those who had smoked fewer than 100 cigarettes in their lifetime, current smokers were identified as individuals who had smoked over 100 cigarettes and were currently smoking, and former smokers were classified as those who had previously smoked over 100 cigarettes but had quit. Alcohol consumption is classified as follows: Never Drinkers (never consumed alcohol), Former Drinkers (no alcohol in the last 12 months), Mild Drinkers (1–3 drinks/week), Moderate Drinkers (up to 1 drink/day for women, 2 for men), and Heavy Drinkers (more than 3 drinks/day for women, 4 for men, or binge drinking) [17,18]. Hypertension is defined as systolic BP ≥130 mm Hg, diastolic BP ≥80 mm Hg [19]. Diabetes was detected by measuring fasting plasma glucose (FPG) levels (FPG greater than 7.0 mmol/L or glycohemoglobin HbA1c (%) higher than 6.5), as well as receiving confirmation from the doctor. The presence of chronic kidney disease (CKD), rheumatoid arthritis, and diabetes was determined by physicians evaluating whether the participants had reported any of these conditions.

### Statistical analyses

The statistical analysis was conducted by including sample weight according to CDC guidelines to account for the complexity of the survey design. The sample weight for NHANES cycles was computed as (1/N) × WTN, with WTN being derived from the variable WTMEC2YR in NHANES 1999–2014 demographic documents [20]. The study population characteristics were summarized by classifying ASCVD into two categories. The mean (95% CI) weighted by the survey was used to present continuous variables, whereas percentages (95% CI) were used to express categorical variables. Survey-weighted linear regression and the Chi-square test were utilized to calculate p-values for continuous and categorical variables, correspondingly.

Subsequently, we performed weighted logistic regression analyses, adjusting for various factors to examine the association between ASCVD and moderate/severe periodontitis. The unadjusted model included no covariates. Model 1 adjusted for gender, age, race, educational level, and PIR, while Model 2 further accounted for BMI, smoking history, alcohol consumption, physical activity, hypertension, and diabetes. A subgroup analysis was also conducted. Finally, we performed an interaction analysis to assess the indirect effects of covariates on the relationship between periodontitis and ASCVD risk. All analyses were performed using R software, and statistical significance was set at *p <* 0.05 (two-sided).

## Results

### Baseline characteristics

**Fig 1:**
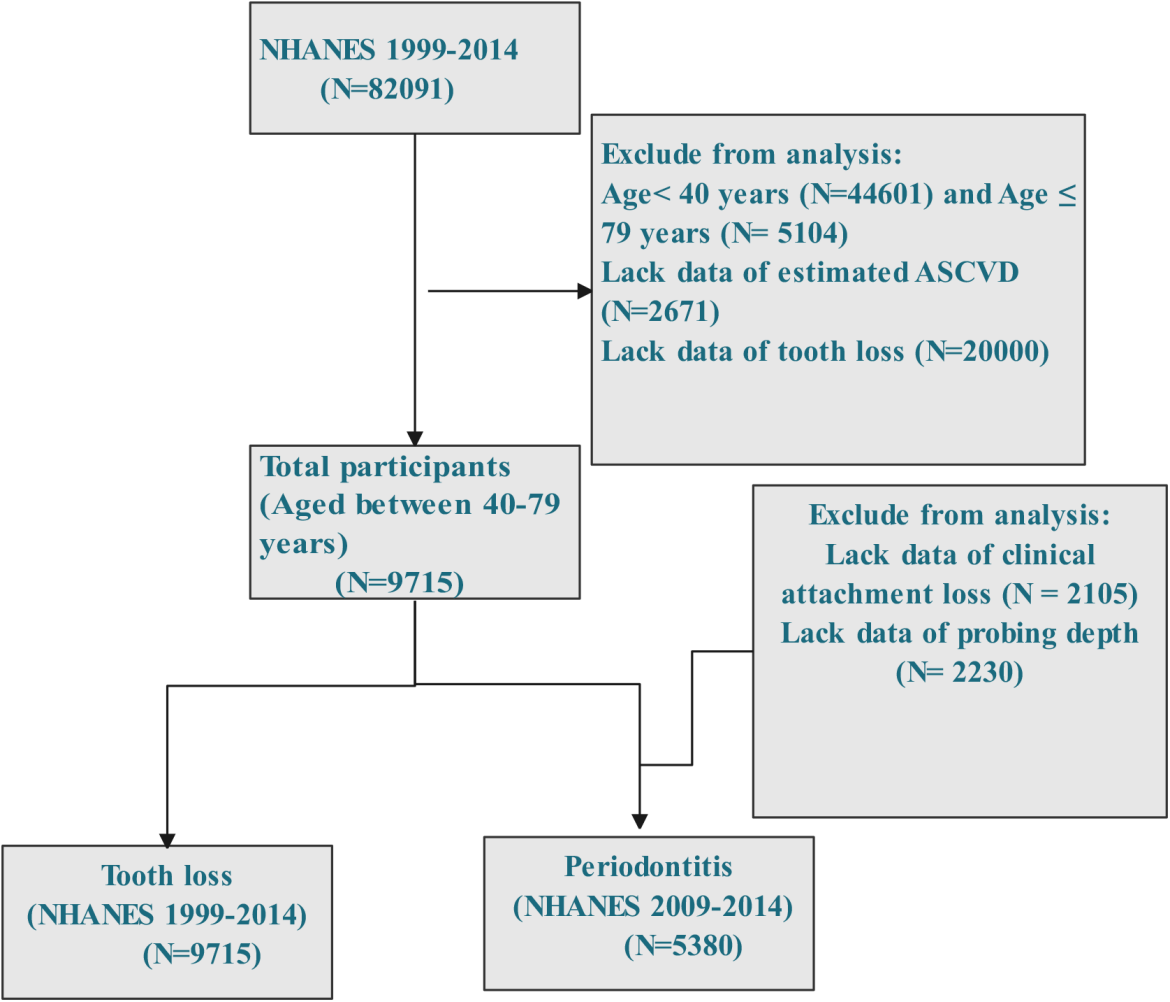
Flowchart of sample selection for NHANES 1999–2014.

The study analyzed data from NHANES 1999–2014, involving a total of 82,091 participants. Subjects who did not have ASCVD risk scores, periodontal examinations, or data on key covariates were excluded from the analysis. The final sample included 9,751 adults (for tooth loss) and 5,380 adults (for periodontitis) aged between 40 and 79 years (Fig 1). Of these participants, more than 56.64% lost 0–4 teeth, 12.91% lost 5–8 teeth, 17.99% lost 9–27 teeth, and 12.46 were edentulous. Out of the total, 28.99% experienced moderate to severe periodontitis. There was a notable disparity in ASCVD rates between genders, with 83.18% of females having a ten-year ASCVD Risk compared to only 16.82% of males. People who had an increased ten-year ASCVD Risk were more likely to be female, older, have a higher BMI, a higher occurrence of CVD, CKD, and hypertension, smoke, diabetes, and consume less alcohol consumption. Table 1 summarizes the baseline characteristics.

**Table 1:** Weighted characteristics of study participants based on ASCVD group.

Variable	Total (n=9751)	Low 10-year ASCVD risk (< 7.5%) (n=5175)	Elevated 10-year ASCVD risk (≥ 7.5%) (n=4576)	P value
**Age**	58.64 ± 11.41	51.33 ± 7.94	66.91 ± 8.79	**<0.0001**
**PIR**	3.08 ± 1.64	3.40 ± 1.65	2.71 ± 1.54	**<0.0001**
**BMI (kg.m2)**	29.02 ± 6.34	28.52 ± 6.18	29.58 ± 6.46	**<0.0001**
**Coffee Intake(gram)**	494.19 ± 635.19	504.69 ± 669.87	482.29 ± 593.34	0.08
**Drinks/day**	2.23 ± 1.96	2.50 ± 2.12	1.82 ± 1.61	**<0.0001**
**Number of Tooth Loss**	7.98 ± 9.70	4.98 ± 7.68	11.38 ± 10.59	**<0.0001**
**Tooth loss group**				**<0.0001**
0–4 lost teeth	5523 (56.64)	3690 (71.30)	1833 (40.06)	
5–8 lost teeth	1259 (12.91)	608 (11.75)	651 (14.23)	
9–27 lost teeth	1754 (17.99)	580 (11.21)	1174 (25.66)	
28 lost teeth	1215 (12.46)	297 (5.74)	918 (20.06)	
**Sex**				**<0.0001**
Female	4762 (48.84)	1839 (35.54)	2923 (63.88)	
Male	4989 (51.16)	3336 (64.46)	1653 (36.12)	
**Marital status**				**<0.0001**
Living with partner	328 (3.40)	230 (4.49)	98 (2.16)	
Married	8756 (90.78)	4501 (87.96)	4255 (93.97)	
Never married	561 (5.82)	386 (7.54)	175 (3.86)	
**Education level**				**<0.0001**
Less than 9th grade	77 (3.49)	28 (2.37)	49 (4.79)	
9-11th grade	253 (11.47)	113 (9.56)	140 (13.69)	
High School graduate	487 (22.09)	241 (20.39)	246 (24.05)	
Some college or AA degree	720 (32.65)	364 (30.80)	356 (34.80)	
College graduate or above	668 (30.29)	436 (36.89)	232 (22.68)	
**CVD**				**<0.0001**
No	8278 (84.90)	4757 (91.92)	3521 (76.96)	
Yes	1472 (15.10)	418 (8.08)	1054 (23.04)	
**CKD**				**<0.0001**
No	7829 (80.88)	4709 (91.45)	3120 (68.86)	
Yes	1851 (19.12)	440 (8.55)	1411 (31.14)	
**DM**				**<0.0001**
No	7239 (74.24)	4372 (84.48)	2867 (62.65)	
DM	1674 (17.17)	361 (6.98)	1313 (28.69)	
IFG	496 (5.09)	271 (5.24)	225 (4.92)	
IGT	342 (3.51)	171 (3.30)	171 (3.74)	
**Menopause status**				0.09
No	234 (8.93)	64 (10.77)	170 (8.39)	
Yes	2387 (91.07)	530 (89.23)	1857 (91.61)	
**Hypertension**				**<0.0001**
No	4789 (49.11)	3345 (64.64)	1444 (31.56)	
Yes	4962 (50.89)	1830 (35.36)	3132 (68.44)	
**Alcohol user**				**<0.0001**
Never	963 (10.14)	319 (6.33)	644 (14.44)	
Former	2112 (22.24)	867 (17.22)	1245 (27.92)	
Mild	3865 (40.71)	2125 (42.20)	1740 (39.02)	
Moderate	1346 (14.18)	848 (16.84)	498 (11.17)	
Heavy	1209 (12.73)	877 (17.41)	332 (7.45)	
**Periodontitis**				**<0.0001**
No	3628 (67.43)	2347 (72.96)	1281 (59.22)	
Mild	192 (3.57)	122 (3.79)	70 (3.24)	
Moderate	1262 (23.46)	568 (17.66)	694 (32.09)	
Severe	298 (5.54)	180 (5.60)	118 (5.46)	
**Smoke status**				**<0.0001**
Never	4283 (43.92)	2446 (47.27)	1837 (40.14)	
Former	3439 (35.27)	1723 (33.29)	1716 (37.50)	
Now	2029 (20.81)	1006 (19.44)	1023 (22.36)	
**Parkinson**				**<0.01**
No	9620 (98.66)	5124 (99.01)	4496 (98.25)	
Yes	131 (1.34)	51 (0.99)	80 (1.75)	
**Arthritis**				**<0.0001**
No	5913 (60.75)	3703 (71.67)	2210 (48.40)	
Yes	3820 (39.25)	1464 (28.33)	2356 (51.60)	

Abbreviation: ASCVD: Atherosclerotic Cardiovascular Disease; BMI: Body Mass Index; CVD: Cardiovascular Disease; CKD: chronic kidney disease; DM: Diabetes Mellitus; IFG: Impaired Fasting Glucose; IGT: Impaired Glucose Tolerance; PIR: Poverty Income Ratio.

### Association of 10-year ASCVD risk with tooth loss

Table 2 illustrates the correlation between ASCVD and tooth loss. The analysis of a continuous variable (β 10.83, 95%CI 9.42–1.66, P=0.0337) and a categorical variable indicates a higher probability of tooth loss (OR 1.16, 95%CI 1.09–1.39, P=0.0248) in the model II. In all three regression models (Models I to II), the number of lost teeth was positively associated with 10-year ASCVD risk (P<0.05). The risk of ASCVD increases as the number of lost teeth rises.

**Table 2:** Association between ASCVD and number of tooth loss in different models.

Exposure	Non-adjusted	Adjust I	Adjust II
**Tooth loss (no vs yes)**			
10-year ASCVD risk			
<0.075	1.0	1.0	1.0
>=0.075	3.70 (3.34, 4.11) <0.0001	2.45 (2.12, 2.83) <0.0001	1.16 (1.09, 1.39) 0.0248
10-year ASCVD risk (continue)	175.94 (104.25, 296.93) <0.0001	24.65 (12.70, 47.83) <0.0001	10.83 (9.42, 1.66) 0.0337
**Tooth loss** (continuous variable)			
10-year ASCVD risk			
<0.075	0	0	0
>=0.075	6.40 (6.03, 6.76) <0.0001	4.25 (3.72, 4.79) <0.0001	0.36 (−0.19, 0.90) 0.1982
10-year ASCVD risk(continuous variable)	17.50 (16.41, 18.59) <0.0001	12.23 (10.61, 13.85) <0.0001	11.80 (10.89, 14.50) 0.0353

Non-adjusted model adjust for: None

Adjust I model adjust for: sex, age, race, education level and marital status.

Adjust II model adjust for: sex, age, race, education level, PIR, BMI, marital status, menstrual status, smoking status, coffee intake, alcohol intake, hypertension, CKD, and diabetes.

In the gender stratification analysis, females were more significantly affected than males (Females: OR 2.610, 95% CI 2.394–2.844, P<0.0001; Males: OR 1.984, 95% CI 1.863–2.114, P<0.0001, Table 3). For individuals aged less than 59 years, the OR was 1.323 with a 95% CI of 1.247 to 1.404 (P<0.0001). For individuals aged 59 years and older, the OR was 1.938 with a 95% CI of 1.797 to 2.091 (P<0.0001). These results suggest a stronger association between age and tooth loss in the older age group (≥59 years) compared to the younger age group (<59 years). People with an elevated BMI encountered a more pronounced impact of tooth loss on the risk of developing ASCVD over a decade. The effect was substantial for individuals with BMI of 25 or greater (OR 2.004, 95% CI 1.830–2.195, P<0.0001) and for those with a BMI less than 25 (OR 1.975, 95% CI 1.867–2.089, P<0.0001). The interaction between BMI and tooth loss was not statistically significant (P-interaction=0.771). Subjects without a history of CKD had a greater likelihood of periodontitis compared to those with CKD (Yes: OR 1.790, 95% CI 1.600–2.002, P<0.0001; No: OR 1.934, 95% CI 1.827–2.047, P<0.0001; P-interaction<0.0001).

**Table 3:** Examining the correlation between ASCVD and number of teeth loss through subgroup analysis.

Covariate	95% CI	P	P for interaction
Sex			< 0.0001
Male	1.984 (1.863,2.114)	<0.0001	
Female	2.610 (2.394,2.844)	<0.0001	
Marital status			0.017
Married	2.018 (1.921,2.121)	<0.0001	
Never married	1.541 (1.254,1.894)	<0.0001	
Living with partner	2.263 (1.772,2.892)	<0.0001	
CKD			0.244
No	1.934 (1.827,2.047)	<0.0001	
Yes	1.790 (1.600,2.002)	<0.0001	
DM			0.55
No	1.711 (1.415,2.069)	<0.0001	
DM	1.842 (1.641,2.068)	<0.0001	
IFG	1.944 (1.833,2.061)	<0.0001	
IGT	1.809 (1.368,2.392)	<0.001	
Hypertension			0.002
No	1.809 (1.698,1.928)	<0.0001	
Yes	2.066 (1.922,2.220)	<0.0001	
Smoking status			< 0.0001
Never	1.579 (1.442,1.730)	<0.0001	
Former	1.977 (1.820,2.148)	<0.0001	
Now	2.469 (2.240,2.721)	<0.0001	
Alcohol user			0.03
Never	1.742 (0.924,1.990)	0.0674	
Former	1.806 (1.643,1.986)	<0.0001	
Mild	2.085 (1.907,2.280)	<0.0001	
Moderate	2.079 (1.812,2.386)	<0.0001	
Heavy	2.319 (1.856,2.897)	<0.0001	
Age group			< 0.0001
<59	1.323 (1.247,1.404)	<0.0001	
≥59	1.938 (1.797,2.091)	<0.0001	
BMI group			0.771
Low (<25)	1.975 (1.867,2.089)	<0.0001	
High (≥25)	2.004 (1.830,2.195)	<0.0001	

### Associations of moderate/severe periodontitis with 10-year ASCVD risk

The connection between moderate/severe periodontitis and an elevated risk of ASCVD is demonstrated in Table 4. When ASCVD is defined as a categorical variable, the risk of periodontitis was linked to the rise in 10-year ASCVD risk in weighted logistic regression analysis. Model 1 demonstrated a correlation with an OR of 2.08, a 95% CI of 1.73 to 2.51, and a p-value of less than 0.0001. Similarly, Model 2 showed an OR of 1.98, with a CI of 1.76 to 2.24 and a p-value of less than 0.0001. In Model 3, the OR was 1.24, with a 95% CI of 1.01 to 1.52 and a statistically significant p-value of 0.0411. When ASCVD is defined as a continuous variable, periodontitis was positively associated with risk of ASCVD (β1.90, 95%CI 1.46–1.75, P=0.0255).

**Table 4:** Association between ASCVD and periodontitis in different models.

Exposure	Non-adjusted	Adjust I	Adjust II
10-year ASCVD risk			
<0.075	1.0	1.0	1.0
>=0.075	2.08 (1.73, 2.51) <0.0001	1.98 (1.76, 2.24) <0.0001	1.24 (1.01, 1.52) 0.0411
10-year ASCVD risk (continuous variable)	4.15 (2.84, 6.05) <0.0001	3.20 (1.77, 5.78) 0.0001	1.90 (1.46, 1.75) 0.0255

Non-adjusted model adjust for: None

Adjust I model adjust for: sex, age, race, education level and marital status.

Adjust II model adjust for: sex, age, race, education level, PIR, BMI, marital status, menstrual status, smoking status, coffee intake, alcohol intake, hypertension, CKD, and diabetes.

According to the analysis, periodontitis had a more significant impact on ASCVD in women than in men (Women: OR 3.116, 95% CI 2.467–3.934, P<0.0001; Men: OR 3.089, 95% CI 2.612–3.654, P<0.0001; P-interaction=0.951, Table 5). The effect of periodontitis on the risk of ASCVD in 10 years was greater in individuals over the age of 59 (OR 1.989, 95% CI 1.560–2.537, P <0.0001; P-interaction< 0.001). Individuals with a higher BMI encountered a stronger impact of periodontitis on the 10-year ASCVD risk. The impact was more significant in individuals with BMI of 25 or greater (OR: 2.365, 95% CI: 1.899–2.946, P <0.0001) in contrast to those with a BMI <25 (OR 1.996, 95% CI 1.749–2.279, P <0.0001). The interaction between BMI and periodontitis was not statistically significant (P = 0.184). Subjects with a history of CKD had a greater likelihood of ASCVD compared to those without CKD (No: OR 1.774, 95% CI 1.345–2.341, P=0.053; Yes: OR 2.043, 95% CI 1.774–2.353, P<0.001; P-interaction= 0.384). Similar findings were observed in Hypertension and DM.

**Table 5:** Examining the correlation between ASCVD and periodontitis through subgroup analysis.

Covariate	95% CI	p	P for interaction
Sex			0.951
Male	3.089 (2.612,3.654)	<0.0001	
Female	3.116 (2.467,3.934)	<0.0001	
Marital status			0.954
Married	2.187 (1.906,2.508)	<0.0001	
Never married	2.304 (1.356,3.916)	0.003	
Living with partner	2.391 (1.172,4.877)	0.019	
CKD			0.384
No	1.774 (1.345,2.341)	<0.0001	
Yes	2.043 (1.774,2.353)	<0.001	
DM			0.338
No	1.901 (0.958,2.180)	0.065	
IFG	1.229 (0.613,2.462)	0.552	
DM	2.036 (1.336,3.104)	0.001	
IGT	3.163 (1.414,7.075)	0.007	
Hypertension			< 0.001
No	1.599 (1.342,1.906)	<0.0001	
Yes	2.554 (2.090,3.123)	<0.0001	
Smoking status			< 0.0001
Never	2.823 (0.98,3.586)	0.087	
Former	1.984 (1.570,2.506)	<0.0001	
Now	1.161 (0.871,1.549)	0.304	
Alcohol user			0.152
Never	2.263 (0.987,3.156)	0.0745	
Former	2.140 (1.599,2.865)	<0.0001	
Mild	2.482 (2.051,3.002)	<0.0001	
Moderate	1.643 (1.153,2.340)	0.007	
Heavy	3.083 (1.949,4.877)	<0.0001	
BMI group			0.184
Low(<25)	1.996 (1.749,2.279)	<0.0001	
High(≥25)	2.365 (1.899,2.946)	<0.0001	
Age group			< 0.001
<59	1.056 (0.816,1.366)	0.675	
≥59	1.989 (1.560,2.537)	<0.0001	

## Discussion

This cross-sectional study examined cross-sectional data from NHANES 1999–2018, involving 9,751 adults (for tooth loss) and 5,380 adults (for periodontitis) aged between 40 and 79 years who were part of a nationally representative sample of American. The main discovery indicated that periodontitis/tooth loss is strongly linked to a higher chance of experiencing major cardiovascular events, regardless of whether the ASCVD risk score was presented as a linear or categorical factor. The correlation remained consistent among all examined subcategories, suggesting that periodontitis could be a potentially modifiable risk factor for ASCVD. Furthermore, the positive association between periodontitis and ASCVD is particularly evident in women, individuals with a BMI of 25 or higher, those diagnosed with hypertension, and frequent smokers or alcohol consumers.

Periodontitis is a condition marked by persistent inflammation of the gums, resulting in the deterioration of the ligaments that hold the teeth and the underlying bone. This inflammatory process can be moderated by various inflammatory mediators, including interleukin-1β, interleukin-6, plasminogen activator inhibitor-1, C-reactive protein, and tumor necrosis factor-α [21,22]. Additionally, these arbitrators have been linked to atherogenic mechanisms and metabolic inflammation, potentially clarifying the association between periodontitis and ASCVD. Notably, chronic periodontitis tissues exhibit stable levels of these inflammatory markers [23,24]. The consensus report by Sanz et al. [25] highlights a clear link between periodontitis and cardiovascular risk, suggesting that periodontitis is a modifiable risk factor for ASCVD. A systematic review supports the idea that periodontal therapy can reduce ASCVD risk [26]. Additionally, Tonetti et al. [27] reinforce this association, emphasizing the importance of early intervention. These references enhance the current analysis by expanding on the cardiovascular implications of periodontal disease. Clinically, periodontitis is characterized by the formation of bacterial biofilms known as dental plaque, which can lead to localized inflammation and activation of host immune responses. Imbalances in the regional microbiota can result in further inflammation and osteoclastic activity, causing alveolar bone resorption [28]. In recent times, microorganisms related to periodontal disease have been linked to infections that can contribute to the onset of systemic inflammation [29,30]. The persistent inflammatory process associated with periodontitis may serve as a key link between oral health and systemic disease.

Gram-negative bacteria in the oral cavity can invade and multiply within endothelial cells, triggering platelet aggregation, blood clot formation, and inflammation—key contributors to atherosclerosis. The host immune response to bacterial products, including lipopolysaccharides (LPS), further intensifies inflammation, leading to elevated levels of pro-inflammatory markers such as C-reactive protein, interleukin-18, and heat shock protein [6,31,32]. These biomarkers are critical risk factors for atherosclerosis development. Bacterial accumulation can lead to bacteremia, transferring lipopolysaccharides into the bloodstream, directly affecting endothelial cells and accelerating atherosclerosis. Therefore, the presence of oral microorganisms, particularly gram-negative bacteria, may significantly influence atherosclerosis progression [33–35].

This study reveals a significant link between periodontitis/tooth loss and 10-year ASCVD risk in non-Hispanic white adults based on NHANES data. The association appears autonomous and consistent. However, due to the cross-sectional nature, establishing causality is not possible. Factors like inflammation markers and tooth brushing habits, missing from the 1999–2014 NHANES data, may have influenced the results. Further research is needed to explore this association, considering a wider range of both recognized and unrecognized variables.

In conclusion, the latest study demonstrated a strong association between periodontitis, tooth loss, and an elevated 10-year ASCVD risk in a representative sample of non-Hispanic white American adults.
